# Aptamer-enriched scaffolds for tissue regeneration: a systematic review of the literature

**DOI:** 10.3389/fbioe.2023.1199651

**Published:** 2023-05-17

**Authors:** Ludovica Parisi, Benedetta Ghezzi, Andrea Toffoli, Guido M. Macaluso, Simone Lumetti

**Affiliations:** ^1^ Laboratory for Oral Molecular Biology, Department of Orthodontics and Dentofacial Orthopedics, University of Bern, Bern, Switzerland; ^2^ Centro Universitario di Odontoiatria, Dipartimento di Medicina e Chirurgia, University of Parma, Parma, Italy; ^3^ Istituto dei Materiali per l’Elettronica ed il Magnetismo, Consiglio Nazionale delle Ricerche, Parma, Italy

**Keywords:** aptamers, regeneration, tissue engineering, tissue scaffold, bioactivity

## Abstract

**Introduction:** Aptamers are a brand-new class of receptors that can be exploited to improve the bioactivity of tissue engineering grafts. The aim of this work was to revise the current literature on *in vitro* and *in vivo* studies in order to i) identify current strategies adopted to improve scaffold bioactivity by aptamers; ii) assess effects of aptamer functionalization on cell behavior and iii) on tissue regeneration.

**Methods:** Using a systematic search approach original research articles published up to 30 April 2022, were considered and screened.

**Results:** In total, 131 records were identified and 18 were included in the final analysis. Included studies showed that aptamers can improve the bioactivity of biomaterials by specific adsorption of adhesive molecules or growth factors from the surrounding environment, or by capturing specific cell types. All the studies showed that aptamers ameliorate scaffold colonization by cells without modifying the physicochemical characteristics of the bare scaffold. Additionally, aptamers seem to promote the early stages of tissue healing and to promote anatomical and functional regeneration.

**Discussion:** Although a metanalysis could not be performed due to the limited number of studies, we believe these findings provide solid evidence supporting the use of aptamers as a suitable modification to improve the bioactivity of tissue engineering constructs.

## 1 Introduction

In the last three decades, tissue engineering (TE) developed as a promising field for the restoration and the regeneration of damaged or lost tissues and organs (Langer and Vacanti, 1993; Berthiaume et al., 2011; Emara and Shah, 2021). Accordingly, TE-grafts have acquired a constant and increasing social value, since they might address the disparity which persists between the limited availability of organ donors and the demanding for transplantation procedures (Shafiee and Atala, 2017). From the very beginning, the TE dogma has relied on the use of a biomaterial-based matrix (scaffold), which act as a template to accommodate cells (Fisher and Mauck, 2013; Parisi et al., 2018). Under opportune stimuli cells proliferate and colonize the scaffold, differentiate, and start to depose new tissue-specific extracellular matrix (ECM). With time, the newly-deposed ECM coat the structure of the temporary graft, which is progressively resorbed until the accomplishment of the regenerative process (Zhang et al., 2014).

**FIGURE 1:**
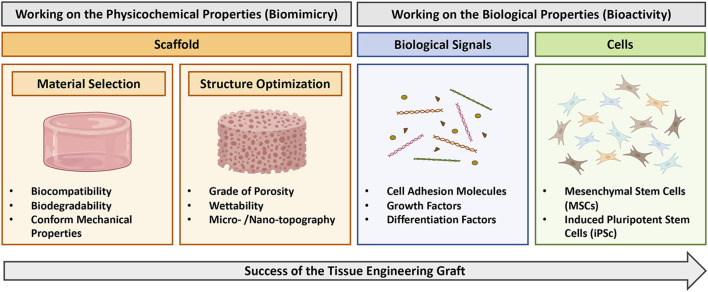
Diagram illustrating strategy to obtain successful tissue engineering grafts. Biomimicry can be address by working on scaffold properties (orange boxes), including selection of the proper material and optimization of the structure. Bioactivity can be obtained by complementing the scaffold with opportune biological signals (blue box) or cells (green box). This figure was realized using Biorender.

To date, much of the progress of TE have been achieved on the development and the optimization of the scaffold structure (Stratton et al., 2016; Parisi et al., 2018). Different type of substitutes, with different mechanical properties, porosities, shapes, micro/nano-topographies and wettability, have been developed and optimized to fulfill different requirements and to reach the maximum level of biomimicry according to the target tissue to restore (Almeida and Bartolo, 2021). However, although they can be effective, a major limitation of TE-scaffolds is their lack of bioactivity, namely, their incapacity to establish an effective crosstalk with the surrounding environment (Williams, 1999; Williams, 2008; Williams, 2022). In more details, bioactivity relies on the combination of proper biological signals and cellular components within the scaffold, which should accelerate and tailor the response of the damaged tissue towards a regenerative process, and therefore lead to an increased possibility of success of the graft itself (Figure 1) (Moysidou et al., 2020).

A quite new approach to enhance the bioactivity of the scaffolding materials involves the promotion of proteins and cells adsorption from the host itself *in vivo* (Guo et al., 2005; Ansari et al., 2013). This might occur by means of receptors, which once immobilized at the biomaterial interface could promote a specific and selective binding of molecules and/or cells from the environment to provide endogenous stimulus to cell colonization and tissue regeneration (Parisi et al., 2017a).

Aptamers are a brand-new class of receptors, which were first discovered in viruses and that became increasingly popular in biomedical research in the past 30 years (Ellington and Szostak, 1990; Tuerk and Gold, 1990). Structurally, aptamers are small single stranded oligonucleotides, which function by recognizing a specific target, thus folding in a highly specialized 3D conformation, and finally binding the target with high and selective affinity (Ellington and Szostak, 1990; Mascini et al., 2012; Ku et al., 2015; Sun and Zu, 2015). Clearly, aptamers resemble the function of monoclonal antibodies (Jayasena, 1999).

To the light of our former efforts (Galli et al., 2016; Parisi et al., 2017b), we believe that the use of aptamers to create selective-binding scaffolds is a promising method to increase the bioactivity of scaffolding materials. However, the use of aptamer-decorated biomaterials for tissue regeneration is still a little explored field in the literature, and a proper review of the studies regarding this topic is warranted. Herein, through a systematic review approach, we selected the studies of the literature, which involve the testing of aptamer-enriched biomaterials *in vitro* and/or *in vivo*. Our aims are: i) to disclose and discuss current approaches to improve scaffold bioactivity by means of aptamers, ii) to recapitulate results on improved cell behavior when in contact with aptamer-enriched materials, and iii) to sum up the studies involving *in vivo* pre-clinical testing.

## 2 Materials and methods

### 2.1 Literature search

An electronic literature search using MEDLINE database was performed. Articles published up to, and including, 30 September 2022 were considered. No language or time restrictions were applied. Grey literature was also searched in opensingle.inist.fr. The electronic search strategy included the following combination of key words, MeSH terms and Booleans operators: aptamer*[tiab] AND (biomaterial*[tiab] OR regenerative medicine [tiab] OR tissue regeneration [tiab] OR tissue engineering [tiab]). Keywords were detected in titles and abstracts. The systematic review was performed according to the PRISMA guidelines (Page et al., 2021).

### 2.2 Inclusion criteria

Studies fulfilling the following inclusion criteria were included in the review: studies considering *in vitro* biological and/or *in vivo* testing of aptamer-enriched biomaterials. The focus was put on the strategy adopted to enrich the bioactivity of biomaterials by aptamers, on cell response to modified biomaterials and on the tissue regeneration outcome.

### 2.3 Exclusion criteria

We excluded studies which did not include *in vitro* biological data or *in vivo* testing, or which concerned the development of aptamer-grafted materials for cancer therapy and diagnostic. We also excluded reviews and commentaries, as well as studies which the full text was not available in English.

### 2.4 Data extraction

We used a standardized data extraction form. Records and titles identified were screened by two authors (L.P. and B.G.) based on the inclusion criteria, and discrepancies were discussed consulting a third independent reviewer (A.T.). Therefore, full texts of the selected abstracts were obtained and included for the final review process. All records were screened, and inclusion was agreed by all the authors. For each study the following items were collected: first author, year of publication, type of aptamer used, *in vitro* outcome and/or target tissue.

## 3 Results

### 3.1 Selection of the studies

**FIGURE 2:**
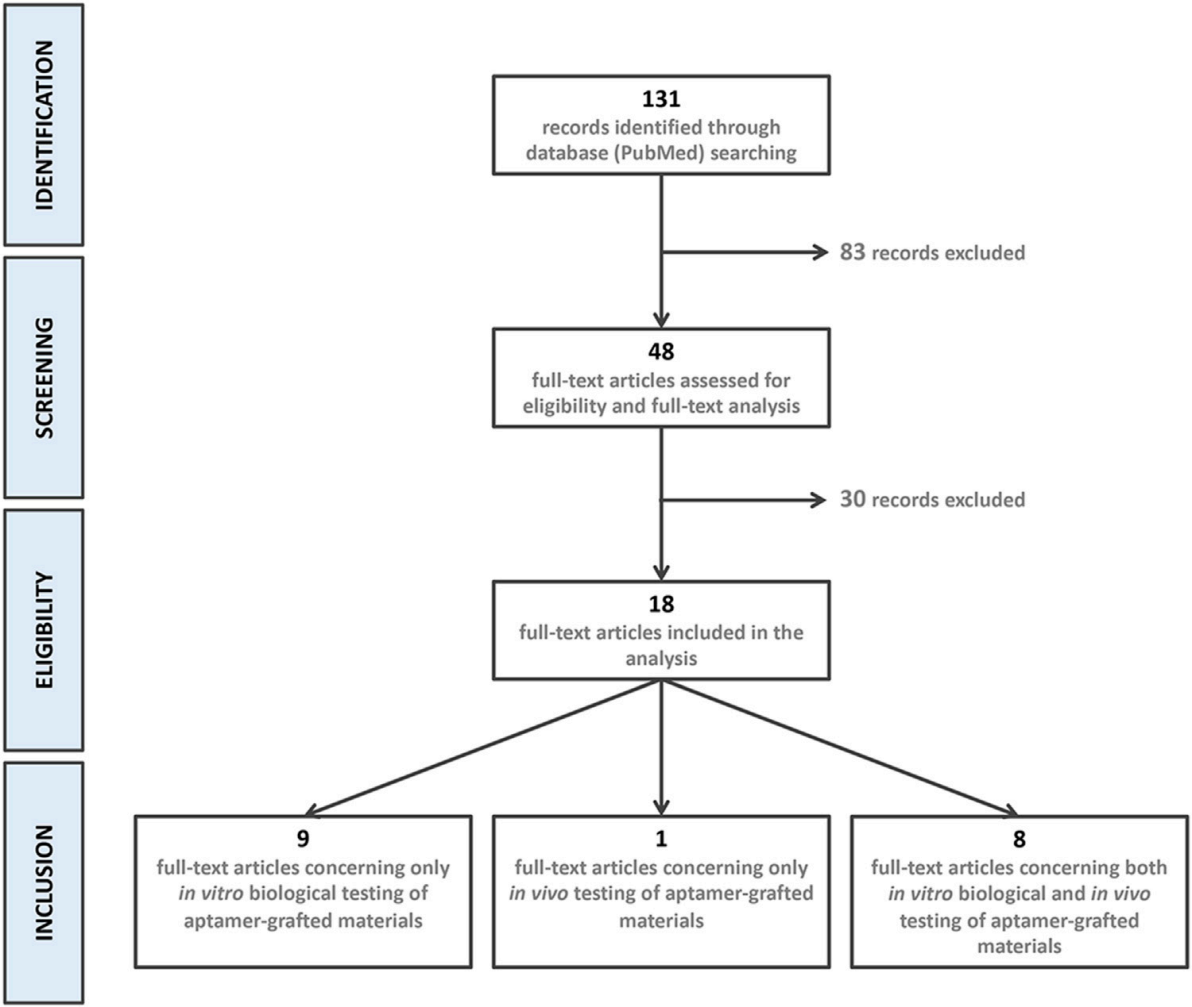
Flow chart of the review strategy.

The electronic search strategy resulted in the identification of 131 references. After title and abstract screening, 83 records were excluded because they did not meet the inclusion criteria. Of the 48 eligible articles, 30 more were excluded after full-text assessment. 18 articles were included in the final analysis: Nine articles were related to only *in vitro* biological testing (Guo et al., 2005; Hoffmann et al., 2008; Chen et al., 2012; Galli et al., 2016; Zhang et al., 2016; Parisi et al., 2017b; Wang et al., 2019a; Parisi et al., 2019; Zhao et al., 2019), 1 to only *in vivo* testing (Enam et al., 2017) and eight presented results of *in vitro* and *in vivo* testing of aptamer-grafted biomaterials (Wang et al., 2017; Zhao et al., 2017; Wang et al., 2019b; Kuang et al., 2019; Kim et al., 2021; Parisi et al., 2021; Sun et al., 2021; Zhao et al., 2021). Records identification, screening, eligibility, and inclusion is presented in Figure 2.

### 3.2 Current approaches to improve scaffold bioactivity by aptamers

**FIGURE 3:**
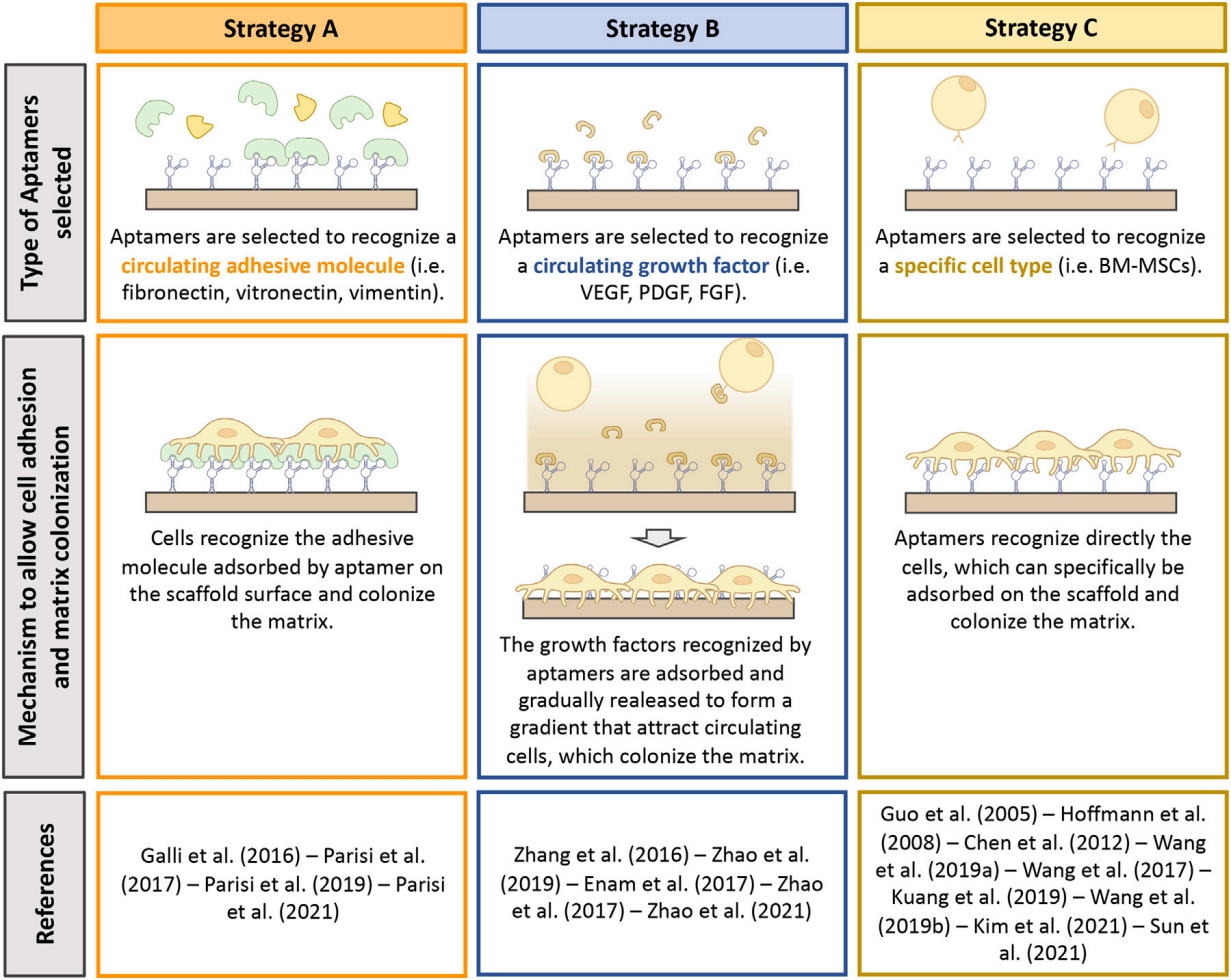
Summary of the strategies adopted to promote scaffold bioactivity by means of aptamers. Type of aptamer selected, and mechanism exploited to improve bioactivity are described. This figure was realized using Biorender.

So far, three different approaches have been proposed to allow the recruitment of specific biological cues on the surface of biomaterials by aptamers. We identified them as Strategy A, B and C (Figure 3). Strategy A was adopted by four studies (Galli et al., 2016; Parisi et al., 2017b; Parisi et al., 2019; Parisi et al., 2021) and concerns the selection of aptamers selected against adhesive macromolecules. After implantation, biomaterials are immediately soaked with patient’s own biological fluid (e.g., blood) and conditioned with proteins contained in it. Because of aptamer presence, specific macromolecules can be adsorbed from the surrounding milieu on the scaffold surface, thus promoting cell adhesion. All the four records considered exploited aptamer selected against fibronectin (FN), a protein that is known to play a key role in cell adhesion (Adams et al., 2015). Similar to Strategy A, Strategy B regards the use of aptamers selected against extracellular molecules, in this case growth factors (GFs). The use of aptamers against GFs offers a double advantage to the design of highly personalized platforms. On one side, the immobilization of specific GFs by means of aptamers contribute to an increase colonization of the scaffold. On the other hand, while cells adhere and colonize the scaffold, the GFs are reversibly released, creating a gradient for the attraction of further cells. Strategy B was proposed by five studies (Zhang et al., 2016; Enam et al., 2017; Zhao et al., 2017; Zhao et al., 2019; Zhao et al., 2021): Three studies used aptamers selected against the vascular endothelial growth factor (VEGF) (Zhang et al., 2016; Zhao et al., 2017; Zhao et al., 2021), one study combined the use of an anti-VEGF aptamer with an anti-platelet derived growth factor BB (PDGF-BB) (Zhao et al., 2019) and one study grafted an aptamer screened for the recognition of fraktalkine (FKN or CXC3CL1) (Enam et al., 2017). Lastly, Strategy C involves the use of aptamers, which recognize specific cell types, allowing selective cell adhesion (Chen et al., 2012; Wang et al., 2019a; Wang et al., 2019b; Kim et al., 2021). This strategy was proposed by eight studies, which used anti-osteoblasts (Guo et al., 2005; Wang et al., 2017), anti-endothelial precursor cells (EPCs) (Hoffmann et al., 2008), anti-T cells (Chen et al., 2012) or anti-mesenchymal stem cells (MSCs) aptamers (Wang et al., 2019a; Wang et al., 2019b; Kuang et al., 2019; Sun et al., 2021). One more study could be included in this category that exploited a CD31-recognizing aptamer, which is a specific marker for endothelial cells (Kim et al., 2021).

### 3.3 Aptamer-enriched biomaterials promote cell behavior *in vitro*

Improved scaffold bioactivity can be obtained at two different levels: i) ameliorating scaffold colonization by cells and ii) promoting their fate and function (Parisi et al., 2018).

17 of the 18 records included in the review investigated *in vitro* cell responses to aptamer-grafted materials (Table 1). Improvement of cell colonization by measuring parameters such as adhesion, migration, viability and proliferation was investigated by all the studies included in the analysis, while increased capacity to control cell fate and function was learned only by five studies.

**TABLE 1:** Summary of the *in vitro* studies.

Work	Year	Aptamer	Strategy			Outcome					
						Colonization				Fate	
			Strategy A	Strategy B	Strategy C	Adhesion	Viability	Proliferation	Migration	Differentiation	Angiogenesis
Guo K et al.	2005	anti-osteoblasts			x	x					
Hoffmann J et al.	2008	anti-EPCs			x	x					
Chen N et al.	2012	anti-T cells			x	x					
Galli C et al.	2016	anti-FN	x				x		x		
Zhang X et al.	2016	anti-VEGF		x		x	x				
Parisi L et al.	2017	anti-FN	x			x		x			
Wang Y et al.	2017	anti-osteoblasts			x				x		
Zhao N et al.	2017	anti-VEGF		x				x			
Parisi L et al.	2019	anti-FN	x			x					
Kuang L et al.	2019	anti-MSCs			x				x		
Wang X et al. (a)	2019	anti-MSCs			x	x		x		x	
Wang X et al. (b)	2019	anti-MSCs			x				x		
Zhao N et al.	2019	anti-VEGF/anti-PDGF-BB		x				x	x		x
Kim DH et al.	2020	anti-CD31			x	x	x			x	
Parisi L et al.	2021	anti-FN	x			x	x			x	
Sun T et al.	2021	anti-MSCs			x		x		x	x	
Zhao D et al.	2021	anti-VEGF		x				x	x		x

To assess proper colonization of the scaffold nine records investigated cell adhesion (Guo et al., 2005; Hoffmann et al., 2008; Chen et al., 2012; Zhang et al., 2016; Parisi et al., 2017b; Wang et al., 2019a; Parisi et al., 2019; Kim et al., 2021; Parisi et al., 2021) and found out a significative increased number of adhered cells in the aptamer groups. Time frame for cell adhesion observations ranged from 4 to 24 h. When aptamers selected against specific cells were used, selective adhesion of the target cell could be investigated (Chen et al., 2012; Wang et al., 2019a; Wang et al., 2019b; Kim et al., 2021). In two cases (Chen et al., 2012; Kim et al., 2021), the use of aptamers was shown to promote the attachment of the target cells and to displace adherence of other cell types. In contrast, Wang et al. (2019a) observed that aptamers selected against MSCs did not impede the adhesion of macrophages and NIH3T3 fibroblasts, but starkly contrasted their spreading. Two studies could further correlate the number of attached cells with the amount of aptamer used for the functionalization (Chen et al., 2012; Parisi et al., 2017b). The other seven studies (Galli et al., 2016; Wang et al., 2017; Wang et al., 2019b; Kuang et al., 2019; Zhao et al., 2019; Sun et al., 2021; Zhao et al., 2021) evaluated proper colonization by assessing cell migration. Yet, the use of aptamers promoted cell migration in all the records considered. To further support the role of aptamers in promoting biomaterial colonization, next to adhesion and/or migration, 10 studies also investigated cell viability or proliferation, (Galli et al., 2016; Zhang et al., 2016; Parisi et al., 2017b; Zhao et al., 2017; Wang et al., 2019a; Zhao et al., 2019; Kim et al., 2021; Parisi et al., 2021; Sun et al., 2021; Zhao et al., 2021). Methods to detect cell viability included stainings (i.e., Calcein-AM or Trypan Blue exclusion assay) and quantification of cell metabolic activity and viability by tetrazolium salts or chemiluminescence assays, respectively. Except for one study (Sun et al., 2021), all the other studies showed that aptamers could support cell viability.

As previously mentioned, amelioration of cell differentiation because of aptamer functionalization was studied from only a minority of studies (5/18%–30%) (Wang et al., 2019a; Zhao et al., 2019; Kim et al., 2021; Sun et al., 2021; Zhao et al., 2021). Three of these studies, which exploited the use of anti-VEGF aptamers (Zhao et al., 2019; Zhao et al., 2021) or of an anti-CD31 (endothelial cell marker) aptamer (Kim et al., 2021), investigated *in vitro* the capacity of the aptamer modification to support new angiogenesis. The two studies that employed the anti-VEGF aptamers observed a facilitated formation of capillary-like structures and a significant increase of endothelial sprouts, after tube formation and sprouting assays, respectively. Additionally, Kim et al. (2021) observed that aptamer modification improved the expression of the vascular tissue-specific markers vascular endothelial cadherin (*VE-Cadherin*) and claudin 5 (*CLDN5*), suggesting the formation of an endothelial structure with tight integrity. Sun et al. (2021), which used an aptamer selected against MSCs observed an improved commitment of the recruited MSCs into osteoblasts, underlined by increase alkaline phosphates (Alp) activity, calcium accumulation in the cultures detected by Alizarin Red and improved expression of the bone-related markers runt-related transcription factor 2 (*Runx2*), *Alp*, osteocalcin (*Ocn*) and osteopontin (*Opn*) during differentiation. Lastly, Wang et al. (2019a) investigated an indirect capacity of the aptamer selected against MSCs to improve cell differentiation. Indeed, the author stated that the benefit of the aptamers relied on the capacity of maintaining MSCs stemness and therefore to increase their trilineage differentiation capacity after specific commitment.

### 3.4 Aptamer-enriched biomaterials sustain tissue regeneration *in vivo*

Still a relative limited number of records used pre-clinical models to investigate whether aptamers are a good modification to support regeneration in diverse tissues (Table 2). The target tissue considered in the literature were bone (four studies) (Wang et al., 2017; Kuang et al., 2019; Parisi et al., 2021; Sun et al., 2021), cartilage (one study) (Wang et al., 2019b), vascular tissue (two studies) (Zhao et al., 2019; Zhao et al., 2021), liver (one study) (Kim et al., 2017) and skin (one study) (Enam et al., 2017).

**TABLE 2:** Summary of the *in vivo* studies.

Work	Year	Aptamer	Strategy			Target Tissue
			Strategy A	Strategy B	Strategy C	
Enam SF et al.	2017	anti-FKN		x		skin
Wang Y et al.	2017	anti-osteoblasts			x	bone
Kuang L et al.	2019	anti-MSCs			x	bone
Zhao N et al.	2019	anti-VEGF/anti-PDGF-BB		x		vascular tissue
Wang X et al. (b)	2019	anti-MSCs			x	cartilage
Kim DH et al.	2020	anti-CD31	x			liver
Parisi L et al.	2021	anti-FN	x			bone
Sun T et al.	2021	anti-MSCs			x	bone
Zhao D et al.	2021	anti-VEGF	x			vascular tissue

#### 3.4.1 Bone regeneration

Two records assessed bone regeneration in a defect operated in the femur condyle of Sprague Dawley rats (Wang et al., 2017; Kuang et al., 2019), one in a critical size calvaria defect of Sprague Dawley rats (Sun et al., 2021) and one in a periodontal fenestration defect operated in Whistar Kyoto rats (Parisi et al., 2021). In all the critical size defect models (Wang et al., 2017; Kuang et al., 2019; Sun et al., 2021), aptamers were proved to support and accelerate new bone formation within the first 4 weeks and to further sustain accomplishment of complete regeneration after 8 weeks. Significantly, aptamers promoted homogeneous bone formation within the entire scaffold, most likely due to a homogenous colonization by the recruited cells. Mature bone phenotype was in all the cases evaluated by micro computerized tomography (µCT) analyzing bone volume and density parameters, as well as confirmed by histological evaluation. Aptamers were found to promote new bone regeneration also in the periodontal fenestration defect, as it was evidenced by µCT analysis and confirmed by the analysis of the cell phenotypes involved in the regenerative process by immunoenzymatic assay (Parisi et al., 2021).

#### 3.4.2 Cartilage regeneration

Potential of aptamers in supporting cartilage regeneration was evaluated by one study (Wang et al., 2019b) in an osteochondral defect created in the knee join of New Zealand white rabbits. Histological evaluation 1 and 2 weeks after surgeries revealed a larger number of cells accommodating in the aptamer-enriched scaffold compared to the non-functionalized group. Furthermore, after 12 weeks of healing the repaired cartilage in the control group resembled the structure of fibrotic cartilaginous tissue, whereas the regenerated cartilage in the aptamer group was similar to the physiological surrounding tissue. These data were further confirmed by specific expression of collagen II in the aptamer group. Notably, cells recruited in the aptamer-enriched group were stained positive for Cd90 and Cd105, two established MSCs surface markers (Dominici et al., 2006), indicating specific recruitment and contribution of these cells in the regenerative process.

#### 3.4.3 Neovascularization

*In vivo* angiogenesis was evaluated subcutaneously in mice. Both the studies that investigated *in vivo* angiogenesis (Zhao et al., 2019; Zhao et al., 2021) observed a substantial contribution of anti-VEGF aptamers in supporting neovascularization. Furthermore, a better engagement of Cd31 and alpha smooth actin (*a*Sma) positive cells was observed in the aptamer group.

#### 3.4.4 Liver regeneration

Anti-CD31 aptamers were exploited by Kim et al. (2021) to improve re-reendothelialization of decellularized scaffolds for liver reconstruction. After *in vivo* implantation in a rat model, aptamer-enriched substrates were observed to limit platelet activation thus avoiding thrombotic lesions and supporting good blood circulation. Reduction of platelet activation was confirmed by reduced *Cd63*, phospholipid scramblase 1 (*Plscr1*) and thrombospondin 1 (*Thbs1*) expression in the aptamer group. Furthermore, when transplanted rats were exposed to fibrotic stimuli, an overall reduction of fibrosis and of *aSma*, vimentin (*Vim*), transforming growth factor ß1 (*Tgfß1*) and metalloproteinase inhibitor 1 (*Timp1*) expression was observed, indicating a protective effect of aptamers against lesion chronicisation.

#### 3.4.5 Skin regeneration

Finally, one study investigated the recruitment and differentiation of immune cells at a skin lesion site in mice by using aptamers selected against fractalkine (FKN) (Enam et al., 2017). *In vivo* analysis demonstrated that aptamers promoted recruitment and migration into the scaffold of Cx3cr1^+^ (fractalkine receptor) cells. Furthermore, FACS analysis of the recruited cells was able to identify a higher fraction of Cd206^+^ cells in the aptamer vs. control group. These results indicate that the use of anti-FKN aptamer is a viable modification to support anti-inflammatory M2 macrophage differentiation and to generate an anti-inflammatory environment prone to tissue regeneration.

## 4 Discussion

The creation of an intimate connection between cells and TE grafts is of utmost importance to succeed proper tissue regeneration. In this arena, a key role is played by scaffold bioactivity, namely, its capacity to establish a proper dialogue with the tissue-resident cells. Herein, we summarized the state of the art on the use of aptamers as surface modifiers to improve the biological properties of TE scaffolds. Indeed, aptamers can be exploited as docking points to enrich scaffold surface with opportune biological stimuli, which in turn can promote cell adhesion and differentiation.

To the present moment three methods have been developed to exploit aptamer-binding properties (Figure 3) and all these methods have been shown to promote material colonization by cells *in vitro* (Table 1) and target tissue regeneration *in vivo* (Table 2). Each of these methods have been demonstrated to possess different strengths. Strategy A, which consists in immobilizing adhesive-molecules-binding aptamers to promote cell adhesion, has been shown to improve the biological activity of the protein adsorbed compared to direct coating (Saccani et al., 2019). Similarly, Strategy B allows a progressive and continuous release of growth factors after their adsorption to prolong and sustain cell recruitment. Lastly, Strategy C has been observed to promote the adsorption of specific cell types, thus guiding selective cell response (Chen et al., 2012; Wang et al., 2019a; Wang et al., 2019b; Kim et al., 2021).

We believe that the use of aptamers to modify the surface of biomaterials and therefore create highly bioactive platforms is important for several reasons.

1) First of all, the use of aptamers involves several advantages compared to other class of molecules, which can be crafted to bind different targets (e.g., monoclonal antibodies) (Keefe et al., 2010). Although expensive, the process to produce aptamers (SELEX) is scalable and sustainable. Indeed, the aptamer binding-capacity can be modulated by increasing or decreasing the number of SELEX cycles for their production (Rosch et al., 2020; Xu et al., 2021). In parallel, differently from monoclonal antibodies selection, aptamer purification does not require the use of animals, which are euthanized at the end of the productive process. Clearly, *in vitro* selection also avoids risks related to viral or bacterial contaminations of the final product (Jayasena, 1999). Furthermore, aptamers can be reversibly denaturated, a fact that would definitely help packaging and transport of a potential final product ready for the market (Jayasena, 1999). Being small single or double-stranded oligonucleotides, aptamers are also invisible to the immune system (Group, 2002; Group, 2003; Ireson and Kelland, 2006). More importantly, aptamers should be devoid of any risk to elicit systemic toxicity. Indeed, although none of the selected studies have investigated this point, the fact that when free in the blood plasma aptamers are digested by endogenous nucleases (Lakhin et al., 2013), let us speculate that if released from the platform where they are anchored, aptamers would be quickly degraded without eliciting any adverse effect in the host. Additionally, our own unpublished data also showed that while chitosan scaffolds enriched with aptamers started to be reabsorbed 7 days after subcutaneous implantation, no accumulation of aptamer in blood plasma and urine could be detected. Of course, this is an important aspect that required to be addressed in the future.
2) When a surface modification is introduced, it is important to understand its effects on the mechanical and physicochemical properties of the native scaffold. Indeed, since the scaffold has already been optimized for fitting specific target tissue requirements, its further modification could affect parameters, such as viscoelasticity, hydrophilicity, porosity and roughness. In turn, this could affect and impair cell response, as well as the predicted tissue regeneration (Prasadh and Wong, 2018). Notably, some of the studies we reviewed investigated this point. Chen et al. performed a complete rheology analysis of polyethilenglycol (PEG) hydrogel with or without aptamers, demonstrating that both storage and loss moduli were let undisturbed by aptamer functionalization (Chen et al., 2012). Three other studies (Parisi et al., 2017b; Zhao et al., 2017; Zhao et al., 2021) investigated how aptamer could modify the morphological aspect of functionalized scaffolds. Again, scaffolds maintained comparable porosity and roughness before and after aptamer binding. It should be also reported that three records investigated the effect of aptamers on the hydrophilicity and swelling capacity of different scaffolds (Chen et al., 2012; Parisi et al., 2017b; Wang et al., 2019a). Although the results were contrasting among the studies, aptamers produced a moderate effect on the wettability of the substrates. However, all the authors concluded that the differences were small and could not be considered significant. Notably, other types of surface modification have shown to alter the properties of the scaffolding materials (Stabenfeldt and LaPlaca, 2011). As such, albeit few studies took into consideration the above-mentioned aspect of aptamer functionalization, we can conclude that aptamer grafting on biomaterials seems to be a viable modification to confer enhanced bioactivity without affecting former scaffold optimization.
3) One of the most important compelling advantages of aptamer-decorated biomaterials is the possibility to tailor their surface with different types of aptamers. More specifically, we believe that scaffolds could be envisaged able to capture different mediators from the patient body fluids and concentrate them where they are needed, to trigger specific and different responses. According to this, an example has already been found in the literature (Zhao et al., 2019). The work by Zhao et al. (2019) is indeed a clear example of how two different aptamers (anti-VEGF and anti-PDGF-ßß) can be immobilized to elicit different but complementary responses after scaffold implantation. VEGF and PDGF-ßß are growth factors, which guide angiogenesis mediated by endothelial cells (ECs) and smooth muscle cells (SMCs), respectively (Greenberg et al., 2008; Gianni-Barrera et al., 2018). Hydrogels enriched with anti-VEGF or anti-PDGF-ßß or with their mixture were injected in the hypodermal layer of the skin of mice, and angiogenesis mediated by ECs or by SMCs was evaluated. After 10 days, the anti-VEGF group showed increased recruitment of CD31^+^ cells (ECs) compared to the anti-PDGF-ßß group, and *vice versa* the anti-PDGF-ßß group improved homing of *a*SMA^+^ cells (SMCs). Notably, when the aptamers were combined, maximum recruitment of ECs and SMCs was observed. These data provide strong evidence on the amelioration of the substrate bioactivity by using different aptamers with various targets. Those evidence let us speculate that the combination of several aptamers might be an important end point in the design of more and more performant substrates for TE.
4) Immediately after positioning, TE grafts are soaked with the patient’s own biological fluids (i.e., blood, saliva). Consequently, proteins and other macromolecules are adsorbed on the surface of biomaterials within milliseconds, making the direct experience of the scaffold surface by cells impossible. A question that has been increasingly raised in the latest years regards the study of cell-biomaterial interactions without considering this initial stage (Vogler, 2012; Parisi et al., 2020; Toffoli et al., 2020). We are prone to believe that the use of aptamers also overcome this issue. Indeed, the rationale behind the use of aptamers itself solve this problem.

We acknowledge that a number of limitations can arise from the present report. As mentioned above, none of the studies that considered aptamer-based platform *in vivo* testing have evaluated the lack of systemic toxicity, which can be elicited by aptamers after scaffold degradation. Another important issue regards the fact that a limited number of studies on this topic is still available in the literature. As a consequence, a proper and solid comparison within the collected records is impossible at the present state of the art.

Despite of the limitations, the overall findings suggest a clear benefit of aptamers in improving cell response on biomaterials *in vitro* and tissue regeneration *in vivo*.

## Data Availability

The datasets generated and/or analyzed during the current study are available from the corresponding author on reasonable request.
